# Psychometric evaluation of the Trust in Science and Scientists Scale

**DOI:** 10.1098/rsos.231228

**Published:** 2024-04-17

**Authors:** Sarah M. Wolff, Glynis M. Breakwell, Daniel B. Wright

**Affiliations:** ^1^ Educational Psychology, Leadership, and Higher Education, University of Nevada, Las Vegas, NV, USA; ^2^ Psychology, University of Bath, Bath, UK

**Keywords:** trust, mistrust, science, risk, psychometrics

## Abstract

Reliable and valid measurement of trust in science and scientists is important. Assessing levels of such trust is important in determining attitudes and predicting behaviours in response to medical and scientific interventions targeted at managing public crises. However, trust is a complex phenomenon that has to be understood in relation to both distrust and mistrust. The Trust in Science and Scientists Scale has been adopted with increasing frequency in large-scale public health research. Detailed psychometric evaluation of the scale is overdue and makes meaningful comparisons between studies that use the scale difficult. Here, we examine the scale’s dimensionality across five separate samples. We find that two factors emerge that are divided by their item polarity. Implications for scale use and trust in science measurement are discussed.

## 1. Introduction

Assessment of public trust in science and scientists plays a critical role in public health research, policy and decision making. Measurement of this kind of trust provides valuable insights into public attitudes, beliefs and behaviours related to public health issues [1,2]. There is a persistent link between public attitudes towards science and public knowledge [3]. If measured accurately, individual- and societal-level trust in science and various associations can be used to predict consequential decision making such as someone choosing to get vaccinated or not. Studies have shown the impact of trust in various areas, including vaccine acceptance, health behaviours, risk perception and science communication [4–7]. Measuring trust in science provides the basis for policy makers to identify areas of low trust, develop targeted communication strategies, allocate resources effectively, address misinformation and foster public confidence in scientific research and health interventions [8].

Regardless of the specific crisis, trust in science has been consistently linked to individuals’ behaviour and adherence to recommended preventive measures during public health emergencies around the world [9,10]. Under such circumstances, public reliance on scientists and health authorities to disseminate crisis guidelines increases, and thus public trust in science becomes more critical. Identifying who is or is not complying with advice provided by the scientific community and under what social, cultural or individual conditions is essential to promoting risk moderation behaviours with success [11]. For this reason, the COVID-19 pandemic spurred renewed interest in the measurement of public trust in various sources of information and perceptions of science. Individuals with higher trust in science were more likely to adhere to social distancing, hygiene measures like hand washing, mask wearing and self-isolation recommendations by various governments [12–15]. Trust in science was also shown to predict better public knowledge about the COVID-19 health crisis [16].

On a global level, most people (at least to some extent) trust scientists to do the right thing [17]. Trust is, however, changeable. Recent data suggest that Americans have grown less confident in scientists compared with before the emergence of the coronavirus [18]. Determining what it really means to trust science is key to comprehending how we can measure it with more accuracy and better interpret relationships with other variables. The extent to which beliefs about science can be adequately captured through a direct measure (e.g. ‘How much do you trust science?’) is uncertain. Employing discrete measures of trust, such as perceptions of trustworthiness encompassing benevolence, integrity or ability, may offer a more precise assessment albeit with potential challenges in interpretation [19,20]. In this sense, measures of trust in science are aimed at capturing trustworthiness (or credibility) perceptions rather than trust in science as a behaviour [21].

Psychometric evaluation of widely used instruments that inform public policies are important. Scientific progress often involves challenging and reassessing established practices to ensure they align with evolving standards and knowledge. The merit of such evaluation lies in ensuring the continued validity and reliability of measurements and invites scrutiny of a construct that may spur forward work in the field. While the fact that others have used a measure may suggest some level of acceptance, it does not guarantee its appropriateness or accuracy across different contexts or populations and this is shown here. As methodologies and standards in psychometrics evolve, re-evaluating instruments help researchers align with current best practices and advances in measurement science. This ongoing scrutiny is essential for maintaining the integrity of research findings and fostering a more robust and reliable foundation for public health interventions.

## 2. Measuring trust in science and scientists

In this section, we will be describing the Trust in Science and Scientists Scale (TISS) [22]. Before discussing the particulars of this scale, we will describe what we mean and do not mean by the two main entities that are part of the survey: science/scientists and trust.

### 2.1. What is science and who are scientists?

While philosophers have debated what is and what is not a science (e.g. [23,24]; for reviews see [25,26]), the TISS asks respondents about their views towards science and scientists. These folk notions will be more influenced by cultural norms than by academic texts. They relate to how science and scientists are depicted in movies and the media, and what topics are taught within science (and sometimes engineering) faculties. Entire disciplines get labelled as science and those working in them as scientists, rather than differentiating the approaches more finely as is the norm in philosophy. Furthermore, within a survey context, how people interpret ‘science’ will depend on the other questions asked prior to them in the survey (e.g. [27]). The TISS is often used within surveys asking about attitudes and behaviours related to specific science-related crises, for example, recycling in relation to the climate crisis and vaccination in relation to COVID-19. The goal of much research using TISS is to examine how variation in the resulting scores is related to these attitudes and behaviours.

The ‘science’ and ‘scientists’ respondents must refer to in their responses to TISS statements are those that come to the respondents’ minds. This presents concern for interpreting the results from the TISS because analysists often will not know what specifically respondents believe science/scientists to be, and there will probably be individual differences both between different samples and within the same sample. This is an issue for most surveys and questionnaires.

### 2.2. Trust as a latent construct

Different nuanced definitions of trust have been produced from research depending on which theories the authors of a paper are trying to fit the construct into [28]. We will review some of this, but it is important to stress that the aim of this article is evaluating the psychometric properties of a scale. In that sense, we rely on how they operationalize trust and how researchers have used this scale. Theories of trust are important for this evaluation, and in the discussion, we note how other approaches can also be taken.

Trust is a topic that has been examined from every social science perspective and from levels of analysis at the systemic [29] to the neurophysiological [30]. While sociologists focus on the position and role of trust in social systems [31] and psychologists have been concerned with the development of the capacity to trust with its implications for personality and well-being, social psychologists have examined the role of trust in social influence, group membership and identity processes [32]. The concept of trust, as a form of social capital and a factor in decision making, is also central to many models in economics.

Defining trust as a latent construct poses several challenges due to its multifaceted nature and the subjective interpretations associated with it. Definitions range from the legal (a trust is an arrangement whereby a person—a trustee—holds property as its nominal owner for the good of one or more beneficiaries) to the archaic (where to trust is to give financial credit to someone or to express a hope or expectation). Commonly, it is used to refer to holding a firm belief in the reliability, honesty, strength or ability of someone or something. Different researchers may emphasize distinct aspects of trust, such as trust as a belief in reliability [33], emotional attachment governed by morality [34] or willingness to be vulnerable [35].

While trust often refers to an individual’s willingness to rely on or to have confidence in another person or entity, trustworthiness refers to the characteristics or attributes of the trustee that make them worthy of trust [21]. Trustworthiness encompasses the perceived credibility, integrity, consistency and competence of the trustee that contribute to their deservingness of trust. Trusting someone or something can be viewed as a process of evaluation. This process is not necessarily based on facts, reason or logic. It may also be based on habits, beliefs or emotions and upon the broader purposes or needs of the evaluator. This process is embedded in and influenced by its social context. What constitutes being worthy of trust is socially negotiated [36,37] and varies across cultures [38,39] and over time [40]. The individual evaluates trust in terms determined by norms and expectations established by their group memberships, history and future objectives.

Suffice it to say, trust is complex. It is a multidimensional, subjective, contextually sensitive and culturally variable construct [28]. This presents challenges to its valid, fair and reliable measurement. Yet it is of substantial research interest to examine both trust and trustworthiness in interpersonal, organizational or societal contexts to explore how trustors’ perceptions of trustworthiness influence their trust judgements and behaviours.

### 2.3. Trust in science and scientists

Measuring trust in science has been approached in multiple ways. National data surveys like the United States’ General Social Survey (GSS) use discrete questions that ask about a respondent’s confidence in science or scientific institutions [41]. Besley & Tiffany [19] call into question the ability of this kind of measure to provide information that might guide new public communications strategies. They consider trust theory posits that individuals’ perceptions of scientists are best understood by considering their views on scientists’ competence (i.e. high ability), honesty (i.e. high integrity) and care (i.e. benevolence) [20]. Measuring a latent construct with only one indicator is possible, but it is generally not recommended due to concerns about reliability and construct validity. In general, using multiple indicators provides a more robust assessment of a latent construct by capturing its different facets or dimensions [42,43].

There are a few existing scales with multiple indicators that attempt to measure trust in science, but one has been adopted with increasing frequency in large-scale public health research: TISS [22]. Versions of the scale have been used in recent years to examine differences in compliance with COVID-19 health behaviour guidelines such as vaccination and social distancing [44], associations with misinformation or conspiratorial narratives [45], and associations with perceived risk and mistrust [46]. The TISS has been translated into multiple languages including Spanish [47], Croatian [48], German [49], French [50], Portuguese [51] and Turkish [52]. The number of TISS scale items used in final scale scores across many of these studies varies. For example, Plohl and Musil [14] dropped seven items to achieve an acceptable unidimensional measurement model. Breakwell *et al.* [51] also drop items to achieve something similar, noting that the full scale indicates multidimensionality. The authors of the Turkish adaptation of the scale drop 11 items and opt for a two-factor model (52).

The original scale consists of 21 items constructed to capture trust as a multifaceted attitude response that is sensitive to contextual influence and guided by both feelings and rational thought [22]. The items are presented in table 1. By the scale’s own name, there are two separate ‘trust-in’s being assessed, that of someone’s trust in science and trust in scientists. Sixteen of the final 21 items contain item wording that relates to trust in scientists while only 5 refer to trust in science. There is limited discussion, however, on differentiating between trust in the two targets.

**Table 1.** Trust in Science and Scientists Scale.

Items
1. When scientists change their mind about a scientific idea it diminishes my trust in their work.^a^
2. Scientists ignore evidence that contradicts their work.^a^
3. Scientific theories are weak explanations.^a^
4. Scientists intentionally keep their work secret.^a^
5. We can trust scientists to share their discoveries even if they don’t like their findings.
6. Scientists don’t value the ideas of others.^a^
7. I trust that the work of scientists will make life better for people.
8. Scientists don’t care if laypersons understand their work.^a^
9. We should trust the work of scientists.
10. We should trust that scientists are being honest in their work.
11. We should trust that scientists are being ethical in their work.
12. Scientific theories are trustworthy.
13. When scientists form a hypothesis they are just guessing.^a^
14. People who understand science more have more trust in science.
15. We can trust science to find the answers that explain the natural world.
16. I trust scientists can find solutions to our major technological problems.
17. We cannot trust scientists because they are biased in their perspectives.^a^
18. Scientists will protect each other even when they are wrong.^a^
19. We cannot trust scientists to consider ideas that contradict their own.^a^
20. Today’s scientists will sacrifice the well-being of others to advance their research.^a^
21. We cannot trust science because it moves too slowly.^a^

Scale taken from [22].

^a^ Indicates the reverse-scored items.

Initial items were developed through discussion and revision by six science, technology, engineering and maths (STEM) faculty and two education faculty during the 2011–2012 school year at a metropolitan university. Included are 9 forward and 12 reverse-scored items. The scale authors note that the choice to include both positively and negatively worded statements was to ‘prevent response sets’ [22, p. 81]. In other words, the intention behind the item-wording choices was to reduce the tendency for respondents to answer survey questions in a consistent pattern (i.e. always selecting positive response options regardless of item content), sometimes called acquiescence bias.

Content validity was assessed through input from five researchers external to the project. Minor item revisions continued through one round of pre-testing with a student sample. Authors report a Cronbach’s alpha of 0.86 for their sample of 301 undergraduate students as evidence of the scale’s reliability. As validity evidence, the authors perform a series of correlations between scale score and demographic characteriistics such as religiosity, political ideology, number of college science classes and years of college. They observe the strength, direction and significance of all of these associations consistent with their hypotheses. Tests of dimensionality are not included in the publication. It is inferred that the authors are assuming a unidimensional structure.

Provided that the theoretical and statistical ground that an instrument is built upon can shift over time, continued evaluation of a scale’s psychometric properties is always warranted. Studies furthering the psychometric evaluation of the TISS, however, have lagged. Tests administered across cultures can exhibit startling amounts of differential item functioning (DIF), which can be made even more complex by language adaptations [53]. Differences in scale scores across cultures are meaningful if the assessment is measuring the constructs the same way. It is clear from the mixed use of items (many use only a subset of the original 21 items) across more recent studies that the TISS may benefit from more nuanced evaluation of its items and structure to aid in the standardization of measurement. For these reasons, we sought to examine the psychometric properties of the scale. We note, in particular, a need to explain inconsistencies observed in the scale’s dimensionality.

### 2.4. Measurement quality

Measurement quality can be confounded by response style, method bias and more [42,43]. One example is how the polarity of items (i.e. ‘I am happy with my job’ versus ‘I am not satisfied with my job’) can lead to misinterpretation of items or misinterpretation of the latent factors underlying the scale [54]. It has been common to include both positively and negatively worded items in self-report scales to minimize acquiescence [55]. This practice is based on the assumption that the items measure the same latent construct. However, researchers have raised concerns about the use of negative (or reverse worded) items in rating scales. Mixing question directions (positive or negative wording of items) may not effectively mitigate acquiescence bias. Rather, respondents may still exhibit a consistent response pattern, either agreeing or disagreeing with items, irrespective of their actual attitudes or beliefs [56].

Negative items may relate to latent variables less well than positively worded items, which may reduce the internal consistency of a scale [57,58]. Negative items can be more challenging to interpret correctly and may not produce the same level of agreeability as positively worded items. This discrepancy can stem from differences in response tendencies or cognitive processes triggered by what is often more complex item wording [59]. Response difficulty of reverse-worded items may thus lead to misresponse [60]. The inconsistency with which people respond to positively or negatively worded items may reflect a proclivity to agree due to this requiring less cognitive effort [61] or relate to participant characteristics like self-perception and linguistic performance [62,63].

Moreover, the inclusion of negative items can have substantial detriments to unidimensional model fit. In many cases, a two-factor model, with factors defined by item wording (positive versus negative), will provide a better fit to data than a unidimensional model [64–67]. One explanation is that respondents may exhibit careless responding to the reverse-worded items, leading to response biases and reduced scale validity [68]. This theory considers that individuals with strong feelings or clear attitudes towards the trait being measured are more likely to respond consistently to both regular and reversed items because it is easier to endorse items that align with their beliefs and reject items that contradict them. On the other hand, individuals who fall in the middle of the trait may feel ambivalent or uncertain, leading them to disagree with both types of items since neither fully reflects their nuanced stance on the trait [69]. Researchers also consider that item extremity (polar opposites) plays a role in whether negation items load on a factor distinct from regular items [70]. Another explanation is that the items represent distinct latent constructs that are not adequately captured by a single scale [65,71].

This presents a couple of issues. First, there are practical scoring concerns for scales composed of mixed valence items. In the case that a two-factor model composed of positive and negative items fits better than a unidimensional model, two scores would be needed and possibly weighted to reflect better scoring accuracy, and this may not have been an original intent of the scale’s design. Second, there are important theoretical concerns: do positively worded and negatively worded items exist on opposite ends of a unidimensional construct or do they make up meaningfully different latent constructs? Supportive arguments exist on both sides. In some cases, the suggestion is that opposite-worded items represent different traits [72,73], while in other cases, it is suggested that this is better modelled as method effects [74,75]. These method effects may be modelled as factors or correlated errors. Yet, in any case, there have been suggestions to abandon reverse-scored negatively worded items altogether, to reword them, or to use different response alternatives [57,76].

The TISS contains about half negatively (e.g. We cannot trust science because it moves too slowly) and half positively worded items (e.g. We can trust science to find the answers that explain the natural world). Is trust in science and scientists to be interpreted as an artefact of item polarity response styles, or is it to be interpreted as two distinct but highly related latent constructs? The substantive meaningfulness of this distinction has important implications both for the scoring of the scale and for future directions of research into the latent construct.

### 2.5. The current study

To test the psychometric properties of the TISS, we gathered pre-existing data from three recent separate studies and also the original scale. The current study addresses the following research questions:

— Is trust in science and scientists, as measured with the TISS, a unidimensional latent construct?
— Does this differ or remain constant across countries?

We hypothesized that the TISS would be multidimensional. Specifically, we expect the scale to exhibit two dimensions that are defined by item polarity. In addition, we hypothesized the structure of the scale would be consistent across the countries in our sample, but that measurement invariance would not hold beyond the configural level. Note that this study was not pre-registered.

## 3. Method

### 3.1. Sample

Data were sourced from four separate studies that contained all 21 items of the TISS. Some of these data are freely available; however, permission for use was granted from all authors in all cases. We consider five separate populations from these data and they are described here:

— *UK Sample* [51]: The UK sample consists of 648 (48.5% male, 50.8% female and 0.8% other) residents of the UK, who were recruited online via Prolific during February–March 2021. The average age of the sample is 32.12  years (s.d. = 10.81).
— *PT Sample* [51]: The PT sample consists of 314 (36.8% male, 63% female and 0.2% other) residents of Portugal, who were recruited online via Prolific in the same study as the UK sample, though they received the survey in Portuguese. The average age of the sample is 37.74 years (s.d. = 14.14).
— *DE Sample* [49]: The DE sample consists of 198 (51% male and 49% female) residents of Germany, who were recruited online via Prolific during January 2021 and received the survey in German. The average age of the sample is 32.15 years (s.d. = 11.47).
— *US 2014 Sample* [22]: The US 2014 sample consists of 344 undergraduate students. Data were provided by the authors and did not contain any demographic information. According to their original manuscript, however, the sample of undergraduate students was approximately 46.2% male, 53.8% female and on average 23.86 years old. The students had taken an average of 4.46 science courses. The survey was administered via pencil and paper.
— *US 2021 Sample* [45]: The US 2021 sample consists of 1014 (48.2% male, 50.8% female and 1% other) US residents, who were recruited online via Prolific during January 2021 as nationally representative based on age, sex, race and ethnicity. The average age of the sample is 45.39 (s.d. = 16.40).

We chose to separate the US samples due to the large span of time between when each was acquired. All included samples (except for the original data) were recruited via Prolific during the same time window (early 2021) to reduce heterogeneity and control for potential biases that could arise due to variations in recruitment methods or changes over time. Prolific [77] is an online participant recruitment platform widely used by researchers for data collection in social science and behavioural research.

### 3.2. Materials

The TISS [22] consists of 21 items measured on a rating scale from 1 (Strongly disagree) to 5 (Strongly agree).

### 3.3. Data analyses

Prior to the main analyses, the correlation matrices were examined and Cronbach’s alpha [78] computed for all samples. We report Cronbach’s alpha because many people interpret it as a measure of how well the scale measures a single construct, though many have discussed concerns with how this statistic is interpreted (e.g. [79]). Given that we are investigating the assumptions of this procedure (e.g. tau equivalence), these should not be interpreted as measures of reliability.

Confirmatory factor analysis (CFA) with maximum likelihood estimation with robust standard errors (MLR in Mplus) was used to test the factor structure of the 21-item TISS across a series of measurement models using Mplus 8.8 Models [80].

The following competing measurement models were tested:

— Unidimensional model in which all items were specified to load onto one Trust in Science factor, with one item fixed to 1 and the factor mean fixed to 0, following a fixed-mean-referent-loading approach to identification (CFA-1).
— Correlated two-factor CFA model in which items were specified to load onto two factors composed of positively and negatively loaded items (CFA-2).
— Bifactor CFA model in which items were specified to load onto a general Trust in Science factor as well as one of two domain-specific factors separated by item polarity, with the relationships between specific and general factors constrained to 0 and the variances for the factors constrained to 1 (Bi-CFA-2).

Models were evaluated for fit based on inclusive consideration of fit indices, as well as the admissibility and theoretical consistency of parameter estimates. As a limited test of exact fit, the *χ* ^2^ test can be oversensitive, especially with respect to sample size and minor misspecifications [81,82]. Here, the following series of fit indices were considered in the evaluation of model fit: the comparative fit index (CFI; 83) and Tucker Lewis Index (TLI) [84]; greater than or equal to 0.900 and 0.950 for acceptable and excellent fit; the root mean square error of approximation (RMSEA) [85]; and its 90% confidence interval (as advocated by Steiger) less than or equal to 0.050 and 0.080 for close and reasonable fit [86], and the standardized root mean square residual (SRMR) less than 0.08 [86]. The Akaike information criteria (AIC) [87], the Bayesian information criteria (BIC) [86] and the sample size-adjusted BIC (SABIC) [88] were also evaluated, lower values indicating better model fit. Uniform evidence of goodness-of-fit across indices is often not always observed. For minimizing both Type I and Type II errors, it is recommended to use a combination of relative fit indexes (CFI or TLI) alongside an absolute fit index (RMSEA or SRMR) [86]. We emphasize that while statistical indices provide valuable information about the fit of a model, they should not be the sole basis for decision making on model retention. Researchers must evaluate the overall coherence and theoretical plausibility of the model in conjunction with the statistical evidence to arrive at a comprehensive assessment of model fit [89].

Factor analytic procedures require a series of judgements that ultimately lead to whether a model is deemed acceptable over and above another. A good model is characterized by various qualities such as interpretability, parsimony and generalizability. However, the pivotal criterion for a good model is its superior performance on key dimensions compared with rival models [90]. This is aligned with the notion that scientific progress is achieved when competing theories are compared, and those that least align with the data are either discarded or modified [91]. The selected model can function as a valuable best-working hypothesis until a superior alternative arises. Model selection criteria were guided by the best recommendations by Preacher and Yaremych [92]. First, competing models were compared and substantial improvement in model fit was considered when changes in CFI and TLI (ΔCFI, ΔTLI) are greater than or equal to 0.01 and changes in RMSEA (ΔRMSEA) are greater than or equal to 0.015 [93,94]. In particular, CFI and RMSEA reflect overall discrepancy and include a penalty for model complexity [95]. In addition to the widely used comparison criteria mentioned, ΔAIC and ΔBIC values are also considered, where the absolute difference is greater than 2 [96]. Given that these indices also favour model simplicity, when the more complex model has lower values, this is an indication that the simpler model may just not be good enough. The comparison criteria above were considered alongside the strength of parameter estimates, model complexity, parsimony and theoretical adequacy in line with guidelines across multiple researchers [89]. Factor loadings were considered: excellent above 0.71; very good between 0.63 and 0.70; good between 0.55 and 0.62; fair between 0.44 and 0.33; and poor below 0.32 [97].

To determine whether the instrument behaves the same way across countries, 13 tests of measurement invariance were conducted along the extended taxonomy of Marsh *et al.* [98]. The UK (*n =* 648) and US 2021 (*n =* 1014) samples were used because these are the most likely to have measurement invariance. These sets of data were both sampled from Prolific.co and the items were in their original English form. The US 2014 sample was excluded from these analyses because these data are 10 years older and pre-COVID-19. The PT and DE samples were excluded due to language translations and sample size. Fit indices across the nested and increasingly restrictive invariance models were evaluated for significant change in fit, evident when ΔCFI/TLI are greater than or equal to 0.01 and ΔRMSEA greater than or equal to 0.015 [93,94]. The more constrained model is supported (measurement invariance is supported at different levels) when there is a non-significant change in model fit and the model fit is adequate.

## 4. Results and discussion

### 4.1. Preliminary analyses

Prior to the main analyses, exploratory data analysis was conducted. The items that needed reverse scoring were reverse scored so that higher scores corresponded to more trust in science. Looking at the means for the 21 items for each of the five samples, 94% had their mean above the midpoint of the scale, and 96% have negative skewness. Thus, people tended to trust science. None were normally distributed data (all *p* < 0.001 with Shapiro–Wilk tests, details provided in electronic supplementary material).

The correlation matrices for each of these datasets are available on the OSF page so that others may replicate our analyses or extend them. Details are discussed in electronic supplementary material. Given the sample sizes, a test of differences among the correlation matrices reached statistical significance, Box’s *M* = *χ* ^2^ (693) = 2547.93, *p* < 0.001. How much correlations vary across the five samples can be operationalized in several ways. One method is to look at the spread of pairwise correlations for the five samples. The largest spread in standard deviations was for the correlation between item 7: ‘I trust that the work of scientists will make life better for people’ and item 21: ‘We cannot trust science because it moves too slowly’. Even for this set, the correlations are mostly of similar magnitude and all positive: 0.486, 0.374, 0.086, 0.388 and 0.442, with the German sample having the lowest correlation. More sets of correlations are shown in the electronic supplementary material, and one conclusion is that while the differences among correlation matrices are significantly different (albeit with large samples), the magnitudes of these are similar.

The items (table 1) can be differentiated in several ways, but two are of most interest to us. First, some require reverse scoring. As discussed in §2, there is much interest in whether reverse-scored items should be considered as involving a separate construct than positively worded items. In addition, within the context of ‘trust’ theorizing, the concepts of distrust and mistrust are not opposites of trust [28], so they may also imply multiple-related constructs. A second distinction is whether the item asks respondents about science or scientists. Only five items did not refer to ‘scientists’ so statistical analyses on these should be viewed cautiously. As such, we report detailed analyses on this distinction in the electronic supplementary material but not in the main text. Briefly, the median correlation within the scientists items was *r* = 0.329, within the science items was *r* = 0.370, and between these sets was *r* = 0.362. CFA models show that a two-factor model using this distinction fits better than a one-factor model, but much less well compared with the two-factor model using whether the items required reverse scoring. The TISS was designed and is used assuming there is just a single construct and was not designed to differentiate these sources of variation.

**Figure 1.**
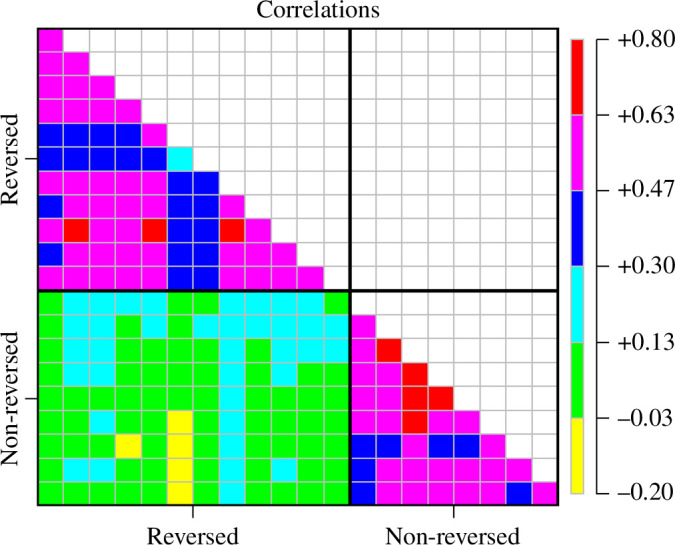
Correlations, for the datasets, partitions with those within the reverse-scored items (top left), within the non-reverse-scored items (bottom right), and between the reverse-scored and non-reverse-scored items (bottom left).

It is worth examining if the correlations within reversed and non-reversed items are larger than the correlations between items that refer to science and scientists. We calculated the mean correlations (using Fisher’s *z* transformation then back-transforming) for the five samples within the reverse-scored items and within the non-reverse-scored items, and between these two sets of the items. For each of the five samples, the mean was lower for the between-sets correlations (around *r* = 0.3) than for the means within the two sets (around *r* = 0.4). This difference is most clearly shown when looking at the correlations between items for all five samples combined. Every correlation between the reversed and non-reversed items is smaller than every correlation within these sets. This is depicted in figure 1. It is important to note that the correlations between reversed and non-reversed items still tend to be positive (98 of 108 or greater than 90%), so it appears that they are measuring different, but related constructs. The authors of the papers that the samples are taken from report Cronbach’s alpha from 0.887 to 0.933. Given that values this high are often interpreted as justifying treating the scale as unidimensional, these exploratory analyses show this can be a bad strategy.

### 4.2. Tests of dimensionality

Model fit statistics for the four competing models across samples are provided in table 2.

**Table 2.** Measurement model fit indices for 21 items.

model	d.f.	*χ* ^2^	RMSEA (90% CI)	CFI	TLI	AIC	BIC	SABIC	SRMR
*UK sample (n = 648)*									
CFA-1	189	891.508	0.076 (0.071, 0.081)	0.840	0.822	31 564.604	31 846.459	31 646.436	0.059
CFA-2	188	461.916	0.047 (0.042, 0.053)	0.938	0.930	31 020.049	31 306.379	31 103.180	0.038
BI-CFA-2	168	399.002	0.046 (0.040, 0.052)	0.947	0.934	30 959.472	31 335.279	31 068.581	0.034
*PT sample (n = 486)*									
CFA-1	189	815.245	0.083 (0.077, 0.088)	0.766	0.740	26 559.413	26 823.144	26 623.186	0.085
CFA-2	188	377.045	0.045 (0.039, 0.052)	0.929	0.921	25 878.425	26 146.342	25 943.210	0.046
BI-CFA-2	168	305.297	0.041 (0.034, 0.048)	0.949	0.936	25 791.177	26 142.819	25 876.207	0.041
*DE sample (n = 198)*									
CFA-1	189	411.764	0.077 (0.067, 0.087)	0.836	0.818	8896.735	9103.896	8904.312	0.063
CFA-2	188	333.608	0.063 (0.051, 0.073)	0.893	0.880	8814.852	9025.301	8822.549	0.057
BI-CFA-2	168	273.556	0.056 (0.043, 0.068)	0.923	0.904	8770.461	9043.387	8780.443	0.051
*US (2014) sample (n = 344)*									
CFA-1	189	739.684	0.092 (0.085, 0.099)	0.711	0.679	14 068.520	14 310.481	14 110.629	0.074
CFA-2	188	471.157	0.066 (0.059, 0.074)	0.852	0.834	13 733.039	13 978.840	13 775.816	0.087
BI-CFA-2	168	324.358	0.052 (0.043, 0.060)	0.918	0.898	13 518.040	13 840.654	13 574.185	0.043
*US (2021) sample (n = 1014)*									
CFA-1	189	1327.006	0.077 (0.073, 0.081)	0.854	0.838	47 467.230	47 777.294	47 577.201	0.051
CFA-2	188	803.029	0.057 (0.053, 0.061)	0.921	0.912	46 778.557	47 093.543	46 890.273	0.042
BI-CFA-2	168	495.604	0.044 (0.039, 0.048)	0.958	0.947	46 386.338	46 799.757	46 532.966	0.030

AIC, Akaike information criteria; BIC, Bayesian information criteria; CFA, confirmatory factor analysis; CFI, comparative fit index; ESEM, exploratory structural equation modelling; RMSEA, root mean square error of approximation, 90% confidence interval in parenthesis; SABIC, sample size-adjusted BIC; SRMR, standardized root mean square residual; TLI, Tucker–Lewis index.

The test of the unidimensional model resulted in a poor fit to the data across all five samples, RMSEA = 0.076–0.092 (90% CI 0.067–0.099), CFI/TLI = 0.679–0.854, SRMR = 0.051–0.085. Compared with the unidimensional models, the correlated two-factor models resulted in substantial improvement in model fit where ΔCFI and ΔTLI were greater than 0.01, ΔRMSEA was greater than 0.015 [93,94], with the exception of the DE sample where ΔRMSEA = 0.014, and absolute values of ΔAIC and ΔBIC were greater than 2 [96]. Good fit of this model was observed across the UK, PT and US 2021 samples, RMSEA = 0.045–0.057 (90% CI 0.039–0.061) and CFI = 0.921–0.938 and TLI = 0.912–0.930. With respect to the standard cut-off values, the correlated two-factor model resulted in a poor fit with the US 2014 [22] and DE samples, albeit these samples are much smaller. It is important to note, however, that the RMSEA tends to favour complex models with high degrees of freedom estimated with large samples and penalize simpler structural equation models that are estimated with fewer variables [99] and analysed with smaller sample sizes [100]. TLI and CFI values for these samples ranged from 0.834 to 0.854.

Bifactor models were also explored for each sample. Results were more varied. The bifactor model resulted in a significant improvement in fit for the DE and US 2014 samples, yet a detriment in fit was observed for the US 2021 sample and a non-significant change was observed for the UK and PT samples. The standardized parameter estimates were ill-defined specific factors across samples, indicating that the bifactor solution is problematic. This could reflect that the loadings on the specific factors are just error, while the general factor captures the commonality shared among the items. The ambiguity in defining specific factors suggests issues with the bifactor model’s ability to accurately represent the underlying structure of the data. In consideration, the correlated two-factor model was retained.

Standardized parameter estimates for the correlated two-factor models across samples are provided in table 3. Factor 1 was generally characterized by appreciable loadings from the reverse-scored items, λ = 0.305–0.784, and Factor 2 was characterized by appreciable loadings from the non-reverse-scored items, λ = 0.333–0.918. Correlations between the factors were moderate to large, positive and statistically significant (*r* = 0.570–0.806). This shows that the factors are highly related, which is why the Cronbach’s alphas were fairly high. It is difficult to interpret the meaning of the factors as something theory related or method related. It may be a combination of the two, and this is discussed further in §5.

**Table 3.** Standardized factor loadings for two-factor CFA solutions across countries (21 items).

Factor—Item #	UK	PT	DE	US (2014)	US (2021)
	λ	λ	λ	λ	λ
Factor 1—1	0.619	0.585	0.448	0.491	0.496
Factor 1—2	0.713	0.713	0.724	0.660	0.736
Factor 1—3	0.697	0.731	0.610	0.576	0.654
Factor 1—4	0.604	0.546	0.672	0.489	0.566
Factor 1—6	0.700	0.698	0.644	0.642	0.740
Factor 1—8	0.376	0.423	0.262	0.459	0.451
Factor 1—13	0.502	0.652	0.508	0.305	0.459
Factor 1—17	0.746	0.784	0.726	0.752	0.795
Factor 1—18	0.615	0.677	0.540	0.573	0.691
Factor 1—19	0.776	0.707	0.572	0.628	0.824
Factor 1—20	0.654	0.639	0.593	0.619	0.649
Factor 1—21	0.681	0.765	0.495	0.579	0.629
Factor 2—5	0.594	0.376	0.500	0.491	0.705
Factor 2—7	0.728	0.634	0.691	0.428	0.775
Factor 2—9	0.773	0.760	0.804	0.806	0.866
Factor 2—10	0.794	0.774	0.710	0.918	0.852
Factor 2—11	0.706	0.738	0.658	0.881	0.727
Factor 2—12	0.710	0.701	0.651	0.508	0.692
Factor 2—14	0.441	0.497	0.660	0.277	0.471
Factor 2—15	0.612	0.611	0.662	0.333	0.649
Factor 2—16	0.557	0.594	0.588	0.300	0.591
Factor correlations	0.757	0.621	0.806	0.570	0.823

*Notes: p* < 0.001 for all estimates. λ = standardized factor loadings.

CFA, confirmatory factor analysis.

### 4.3. Measurement invariance

To examine the extent to which the correlated two-factor model replicated beyond random error, tests of measurement invariance were conducted along the extended taxonomy of Marsh *et al.* [98]. Marsh *et al*. provide a good introduction to these measures and direct readers to further references. Step-by-step guides to testing measurement invariance with various software are provided by van de Schoot *et al.* [101]. A total of 13 partially nested models were tested that involved different combinations of model constraints. Fit indices and model comparisons for these are provided in table 4. Invariance continues to be supported when more restricted models result in a non-significant change in model fit, ΔCFI/TLI are less than or equal to 0.01 and ΔRMSEA is less than or equal to 0.015 [93,94,102].

**Table 4.** Tests of multiple group measurement invariance for the two-factor CFA across country (UK and US 2021).

model	constraints	*χ* ^2^/d.f.	RMSEA	CFI	TLI	ΔRMSEA	ΔCFI	ΔTLI	comparison model
M1-S (configural)	—	1380.448/378	0.056 (0.053, 0.060)	0.918	0.909	—	—	—	—
M2-S (weak)	FL	1612.435/399	0.060 (0.057, 0.064)	0.901	0.896	+0.004	−0.017	−0.013	M1-S
M3-S	FL, Uniq	1647.773/420	0.059 (0.056, 0.062)	0.900	0.900	+0.003	−0.018	−0.009	M1-S, M2-S
M4-S	FL, FVCV	1612.723/400	0.060 (0.057, 0.063)	0.901	0.896	+0.004	−0.017	−0.013	M1-S, M2-S
M5-S (strong)	FL, Inter	1745.757/420	0.062 (0.059, 0.065)	0.892	0.892	+0.006	−0.026	−0.017	M1-S, M2-S
M6-S	FL, Uniq, FVCV	1650.929/421	0.059 (0.056, 0.062)	0.900	0.900	+0.003	−0.018	−0.009	M1-S – M4-S
M7-S (strict)	FL, Uniq, Inter	1778.849/441	0.060 (0.058, 0.063)	0.891	0.896	+0.004	−0.027	−0.013	M-1-S, M2-S, M3-S, M5-S
M8-S	FL, FVCV, Inter	1747.126/421	0.062 (0.059, 0.065)	0.892	0.892	+0.006	−0.026	−0.017	M1-S, M2-S, M4-S, M5-S
M9-S	FL, FVCV, Inter, Uniq	1783.284/442	0.060 (0.058, 0.063)	0.891	0.896	+0.004	−0.027	−0.013	M1-S – M8-S
M10-S (latent mean)	FL, Inter, FMn	1492.943/416	0.056 (0.053, 0.059)	0.912	0.911	+0.000	−0.006	+0.002	M1-S, M2-S, M5-S
M11-S (manifest mean)	FL, Uniq, Inter, FMn	1530.671/437	0.055 (0.052, 0.058)	0.911	0.914	−0.001	−0.007	+0.005	M1-S, M3-S, M5-S, M7-S, M10-S
M12-S	FL, FVCV, Inter, FMn	1747.126/421	0.062 (0.059, 0.065)	0.892	0.892	+0.006	−0.026	−0.017	M1-S, M2-S,M4-S – M6-S, M10-S
M13 -S (complete)	FL, FVCV, Inter, Uniq, FMn	1783.284/442	0.060 (0.058, 0.063)	0.891	0.896	+0.004	−.026	−.013	M1-S – M12-S

CFI, comparative fit index; FL, factor loadings; FMn, factor means; FVCV, factor variance-covariances; Inter, item intercepts; RMSEA, root mean square error approximation and 90% confidence interval in parentheses; TLI, Tucker–Lewis Index; Uniq, item uniquenesses; *χ* ^2^/d.f., chi-square/degrees of freedom ratio.

Here, the configural model in which no constraints were placed on any parameters provided an acceptable fit to the data, RMSEA = 0.056 (90% CI = 0.053, 0.060), CFI = 0.918, TLI = 0.909. This suggests that the construct has the same structure or pattern of free and fixed loadings across the two countries and is the least stringent type of invariance. Subsequently, more restricted models provided significant detrimental fit changes. The weak (metric) invariance model performed significantly worse compared with the configural model, indicating non-equivalence of factor loadings, ΔTLI = −0.013, ΔRMSEA = +0.004, ΔCFI = −0.017. This metric non-invariance shows that the differences in the latent variables are not translated in the same way into differences in the indicators. Examination of the loading patterns revealed that, in general, the UK sample had more appreciable loadings. Partial invariance models could be explored, in which some factor constraints are relaxed one by one until a partially invariant model is achieved [103,104]. The stepwise process of relaxing constraints one at a time post hoc, however, is criticized. It is likely to capitalize on chance and has been dubbed, among other things, a fishing expedition [105,106]. Meaningful comparisons of the variance–covariance matrices cannot be made in cases of metric non-invariance [107]. Strong invariance was not supported, indicating that item intercepts across samples, as well as factor loadings were non-invariant, indicating a likely presence of DIF and therefore no justification for latent mean differences.

## 5. Conclusion

Accurate measurement of trust in science and scientists is crucial for understanding the relationships among public attitudes, beliefs and behaviours in the context of public health and other crises where scientific evidence is important. Such measurements can inform targeted interventions, address disparities in access to healthcare and emergency services, and guide the development of evidence-based policies and communication strategies [2,8]. Through trust measurements, policy makers can promote healthy behaviours and lessen risky ones, foster public support for science-informed policies, and ultimately contribute to outcomes for individuals and their societies.

Analysis, across five samples, showed that a two-factor model offers a better description of the associations than a one-factor solution. We examined if these factors corresponded with those requiring reverse scoring or not, which as discussed above is often identified in psychometric research. Within trust theories, this might also relate to comparing trust with distrust or mistrust. Our emphasis here is on showing that this item differentiation fits, rather than explaining what the factor is. Either way, the TISS was not designed for this purpose. We examined other possibilities, for example, whether the dimensions corresponded with trust in science versus trust in scientists. Detailed analyses are reported in the electronic supplementary material, but the two main points of these analyses were: (i) these models did not fit as well as those using item polarity to differentiate the factors, and (ii) there are only five items in the TISS that do not explicitly mention ‘scientists’ so readers should be cautious interpreting these results. Given that theories of trust differentiate the target of trust and differentiate trust from mistrust and distrust, it would be worth future scales balancing the number of these different types of items, being careful with the use of reverse scored items.

It is apparent both in the correlations (figure 1) and in the CFA that the two-factor solution provides a better fit than the one-factor solution that has been assumed both by the original authors [22] and subsequent researchers using this measure. This two-factor solution is more similar between the US 2021, UK and PT samples in this article. The two-factor solution did not provide adequate fit to the US 2014 and DE data, however. In the case of the US 2014 sample, this might be explained by the context in which the data were collected (pre-COVID) and the more homogeneous population of undergraduates. There were also differences in mode of data collection, which changes the sampling frame. The DE sample was the smallest sample included in these studies.

Tests of measurement invariance revealed that the factor structure (i.e. the pattern of free and fixed loadings) was the same for the UK and US 2021 samples. However, the way people in the two countries respond to the questions is not exactly the same. This suggests that there are some differences in how the trust questions are understood or interpreted between the UK and US 2021 samples. These data suggest that, in general, people in the UK tend to show stronger associations between their responses and their trust in science and scientists compared with those in the USA. With these findings, we caution against assuming measurement invariance without proper testing and validation with given samples. Cross-cultural comparisons should be approached with an assumption that there may be differences in interpretation or response patterns across groups and consider the implications of such biases on the validity and generalizability of their findings. This is further emphasized when also considering language translations.

What the two factors are is worth further discussion. The methods literature discusses that respondents may approach reversed items differently from other items, and this factor could be considered a separate methods effect. However, the trust literature notes how distrust and mistrust are different from each other, and not the opposite of trust. Thus, there may be a separate substantive factor, though this conclusion cannot be drawn with this scale. With the current data, we cannot differentiate these possibilities, but we encourage researchers to estimate both of these factors and see how they relate to other variables. Considering these complexities, we suggest researchers exercise caution when assuming a unidimensional trust in science and scientists variables, as measured with the TISS.

### 5.1. Limitations

This research was not pre-registered. We acknowledge the potential risk of bias introduced by data-dependent analysis and interpretation. To mitigate this potential, we have clearly documented and transparently reported all aspects of the study, including the research questions, hypotheses, data collection procedures, all code used and analysis methods. The present study holds value for several reasons. First, our examination of the TISS across diverse samples contributes valuable insights into its dimensionality. This analysis addresses an existing gap in the literature, offering a nuanced understanding that can inform future research on considerations towards trust measurement. Second, our findings have direct relevance to large-scale public health studies using this scale (or others), providing researchers and practitioners with insights into potential factors influencing trust assessment. By transparently addressing limitations of measurement, we contribute to the ongoing discourse on trust in science, emphasizing the need for methodological improvements in future research. This evaluation addresses the need for transparency and methodological rigour in scale usage. The insights gained from evaluating scales contribute to the standardization of measurement approaches and highlight the need for careful measurement work as a precursor to any large-scale investigation, despite a scale’s previous performance or widespread use.

### 5.2. Future research

The results of this study highlight the value in continued measurement evaluation of existing scales, in particular in the area of trust in science. While reverse-scored items often produce different response patterns and therefore multidimensionality [108], we recognize that the trust concept, as discussed by psychologists, is also multidimensional [28]. Further scale development on the topic should also take into account the different ways in which lay people conceptualize the meaning of science. In some research contexts, it may be beneficial to specify what respondents should be referring to (e.g. medical professionals).

It is also important to examine the wording of different items. Our findings support the suggestion of other studies [57,62,109] to eliminate the use of negatively worded items that is based on a false assumption that they are measuring the same thing as the positively worded items. At minimum, we encourage scale developers not to add negations to question stems as this can complicate how respondents interpret the item and lead to misunderstandings.

## Data Availability

Data and code are available online at OSF [110]. Included are subsets of each dataset containing the items from the TISS, as well as data files prepared for Mplus analyses and correlation matrices for each dataset. Permission was granted from the authors to provide these item data here for transparency and reproducibility. Full datasets for Breakwell *et al*. [51], Jaspal *et al*. [49] and Nadelson *et al*. [22] are not provided. For Agley *et al*., full datasets can be freely accessed at [45]. Electronic supplementary material is available online [111].
